# Author Correction: Synthetic augmentation of cancer cell line multi-omic datasets using unsupervised deep learning

**DOI:** 10.1038/s41467-025-56686-0

**Published:** 2025-02-04

**Authors:** Zhaoxiang Cai, Sofia Apolinário, Ana R. Baião, Clare Pacini, Miguel D. Sousa, Susana Vinga, Roger R. Reddel, Phillip J. Robinson, Mathew J. Garnett, Qing Zhong, Emanuel Gonçalves

**Affiliations:** 1 ProCan®, Children’s Medical Research Institute, Faculty of Medicine and Health, The University of Sydney, Westmead, NSW Australia; 2 INESC-ID, 1000-029 Lisboa, Portugal; 3 Instituto Superior Técnico (IST), Universidade de Lisboa, 1049-001 Lisboa, Portugal; 4 Wellcome Sanger Institute, Wellcome Genome Campus, Cambridge, CB10 1SA UK

**Keywords:** Data integration, Machine learning, Cancer

Correction to: *Nature Communications* 10.1038/s41467-024-54771-4, published online 29 November 2024

In the version of the article initially published, the *y*-axes labels in Fig. 2a were “MOVE reconstruction (pearson’s r)” and have now been corrected to “MOSA reconstruction (pearson’s r)” in the HTML and PDF versions of the article.

