# Supplementary material for: Synthetic augmentation of cancer cell line multi-omic datasets using unsupervised deep learning
Source: Nat Commun. 2024 Nov 29;15:10390. doi: 10.1038/s41467-024-54771-4 (PMC11607321; doi:10.1038/s41467-024-54771-4)
Supplement: Supplementary file 1 — Supplementary Information [file 41467_2024_54771_MOESM1_ESM.pdf]

Supplementary Information

Synthetic augmentation of cancer cell line multi-omic datasets using unsupervised deep learning

Cai et al.

Supplementary Figures

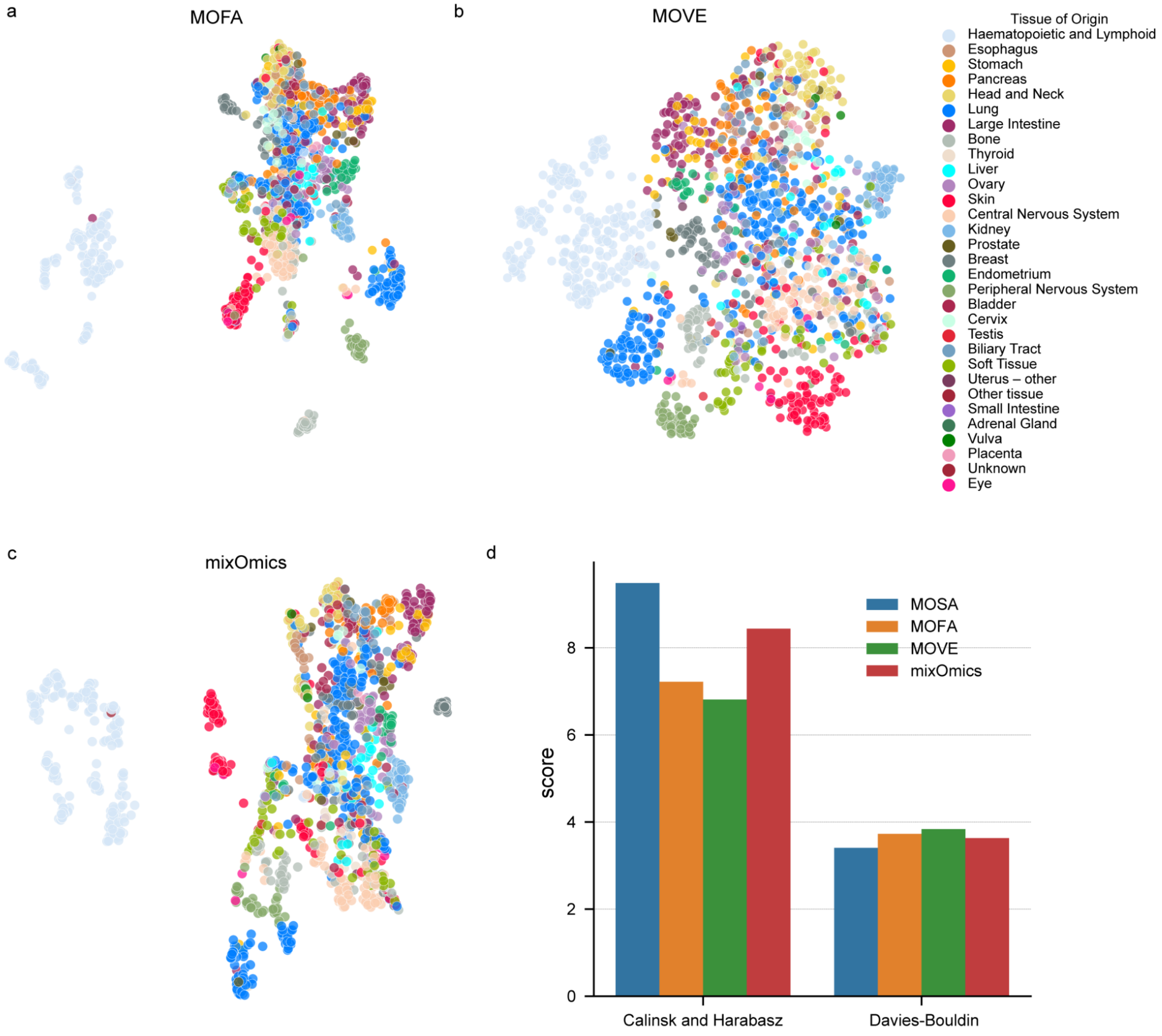

Supplementary Figure 1. *Latent space visualization comparison.* ***a)*** *UMAP representation of the trained MOFA joint latent space, where each dot represents a cancer cell line and is coloured according to its tissue of origin.* ***b)*** *and* ***c)****, UMAP representations for MOVE and mixOmics, respectively.* ***d)*** *comparison of cell line separations quantified by Calinski-Harabasz index (higher value indicates better) and Davies-Bouldin index (lower value indicates better).*

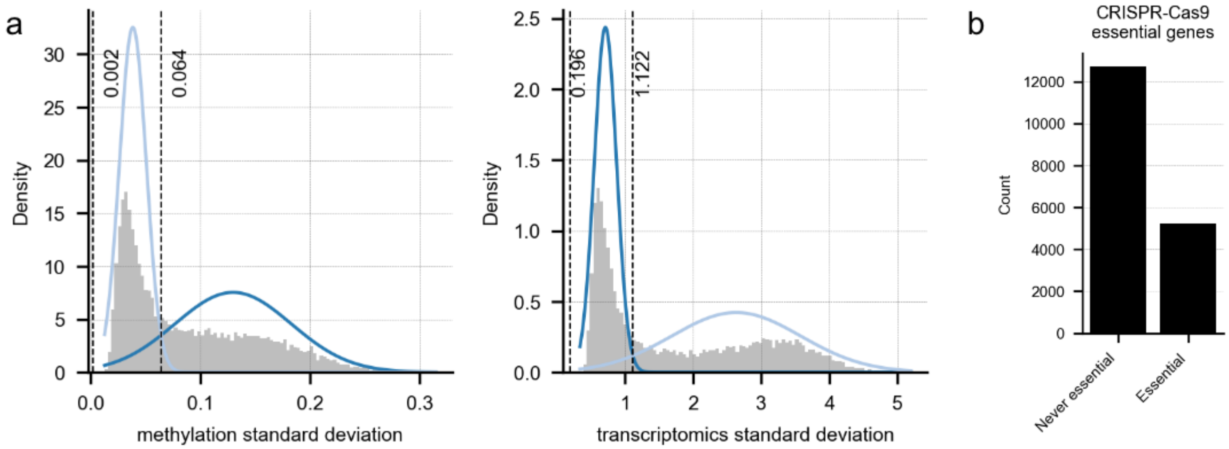

Supplementary Figure 2. *Feature filtering in MOSA.* ***a)*** *gaussian mixture model (k=2) trained to capture gene-promoter methylation (left panel) and gene expression (right panel) features that are lowly variable. Standard deviation thresholds at which the Gaussian mixtures have the same height, i.e., the probability of feature belonging to either distribution is the same, are represented by vertical lines. The rightmost threshold is used for feature selection for the encoders, specifically those that have a standard deviation smaller than the rightmost threshold are masked.* ***b)*** *CRISPR-Cas9 screens were scaled using previously reported essential and non-essential genes (see* ***Methods****), and genes with a scaled gene essentiality score lower than 50% of the median of essential genes (-1) were marked as 1, otherwise 0. Never essential genes denote those genes that were never found to have a scaled gene essentiality lower than the median of the essential genes, and therefore were filtered for the encoders.*

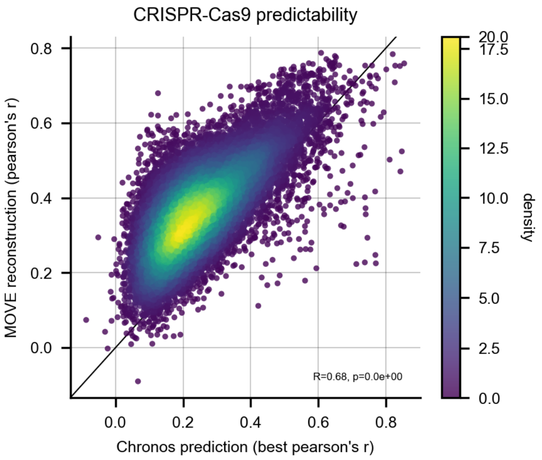

Supplementary Figure 3. *Comparison of MOSA's reconstruction quality score against Chronos DepMap supervised prediction quality score calculated similarly (i.e., 10-fold cross-validation and Pearson's correlation between reconstructed and measured)[34]. For each gene, Chronos estimates three models (Core Omics, DNA-based and Related), and for this comparison, the best Pearson's r score per gene was selected.*

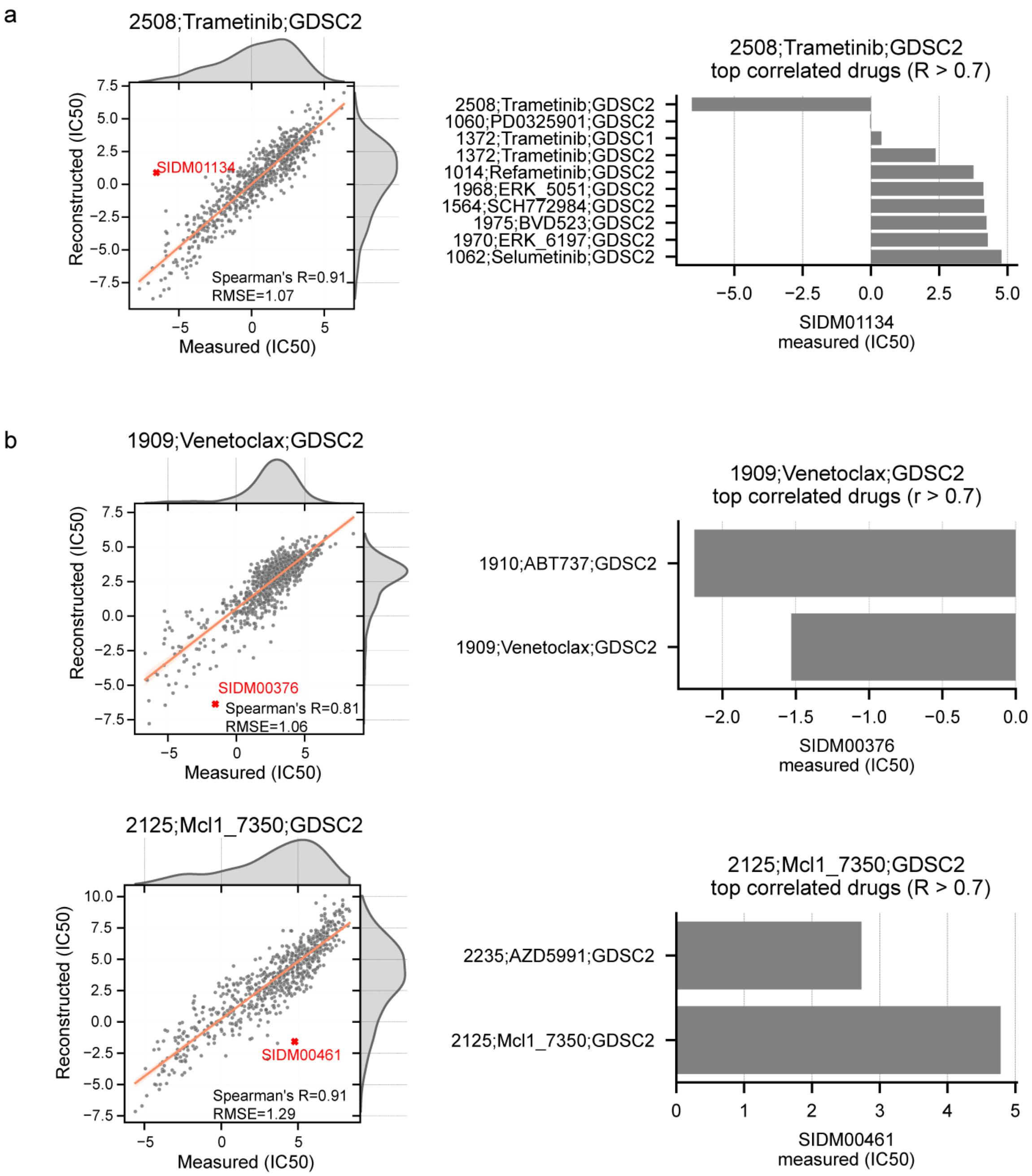

Supplementary Figure 4. *Drug response reconstruction benchmark.* ***a)*** *the biggest absolute difference between the reconstructed drug response data and the original dataset. In the left panel, a regression plot between the reconstructed drug response and the original measurements is shown, with the particular cell line, where the highest discrepancy was observed highlighted with a red cross. In the right panel, a barplot of the measured IC50 of the highlighted cell line is shown. The drugs were selected according to their IC50 correlation to the drug with the biggest discrepancy.* ***b)*** *similar to c), in this case, two representative drugs that target the antiapoptotic MCL1/BCL2 pathway were chosen and plotted among those with the highest discrepancies.*

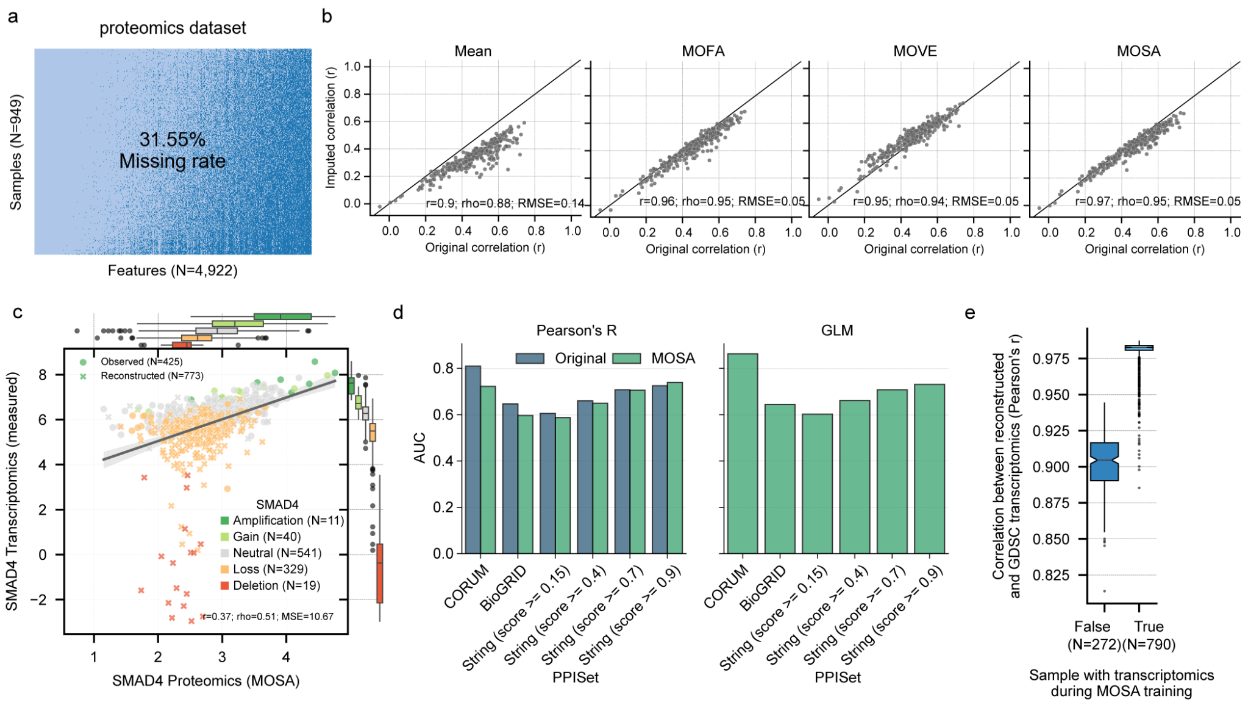

Supplementary Figure 5. *Proteomics missing value reconstruction.* ***a)*** *representation of missing values present in the proteomics dataset, where missing values are represented by dark-blue cells in the matrix, and observations with light-blue.* ***b)*** *comparison of the original (i.e., used for training MOSA) and imputed datasets with an independent proteomics dataset[38]. Each dot represents a cancer cell line present in both the original and independent proteomics dataset and the correlation (Pearson's r) between the two for every cell line is reported in the x-axis. In the y-axes the correlations are reported for the same cell lines, between the imputed matrices using the different methods and the independent dataset.* ***c)*** *correlation between measured SMAD4 gene expression and synthetic SMAD4 protein intensity by MOSA. Circle represents cell lines with original proteomic data (Observed) and cross represents cell lines with synthetic proteomic data (Reconstructed). Box-and-whisker plots show 1.5 x interquartile ranges, centers indicate medians.* ***d)*** *Bar plot of the area under the curve (AUC) of the recall curve when recapitulating protein-protein interactions (PPIs) present in a certain set. The PPI sets used are those from CORUM[76], BioGRID[77] and STRING[78] with 4 different score thresholds. The AUC was calculated using the p-value of either Pearson's R correlation coefficient (left) or the effect sizes of the generalized linear model (right)[40]. This last method cannot be computed on the original data since it has missing values.* ***e)*** *correlation of MOSA's reconstructed transcriptomics against an independently processed transcriptomics dataset. A total of 1,062 were considered in this analysis, from which 272 cancer cell lines did not have any transcriptomics for training MOSA.*

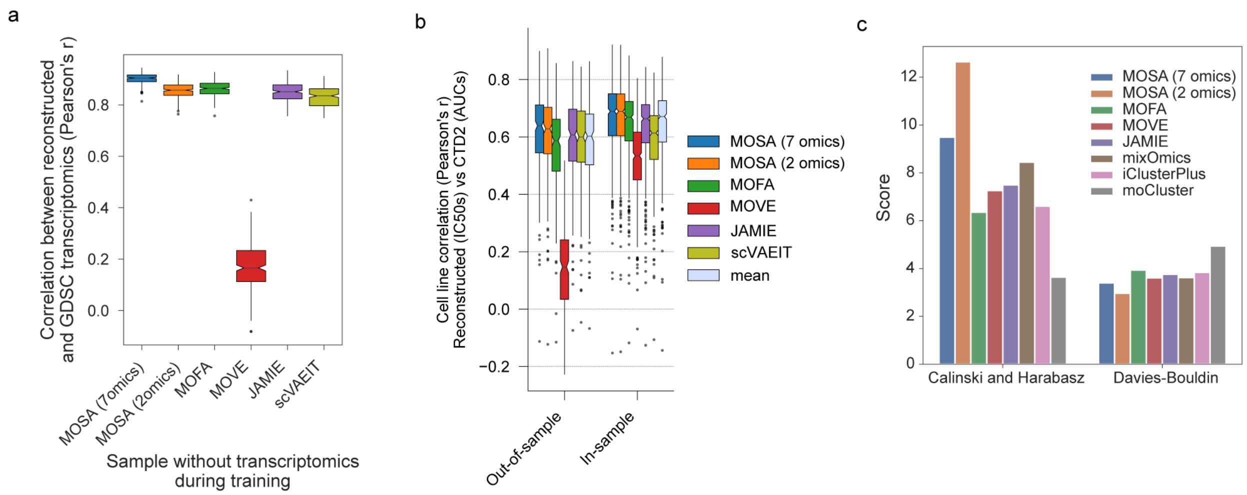

***Supplementary Figure 6**. MOSA benchmark against two omics integration. **a)** correlation of reconstructed transcriptomics against an independently processed transcriptomics dataset. **b)** cancer cell line correlations (Pearson's r) between an independent drug response dataset (CTD2[5,6]) and the MOSA reconstructed dataset, grouped by whether the cancer cell line had prior availability of drug response in the datasets for the model training versus cell lines without drug response data. **c)** tissue of origin comparison of cell line separations quantified by Calinski-Harabasz index (higher value indicates better) and Davies-Bouldin index (lower value indicates better). For better visualization, scVAEIT is not included in this comparison due to its suboptimal performance.*

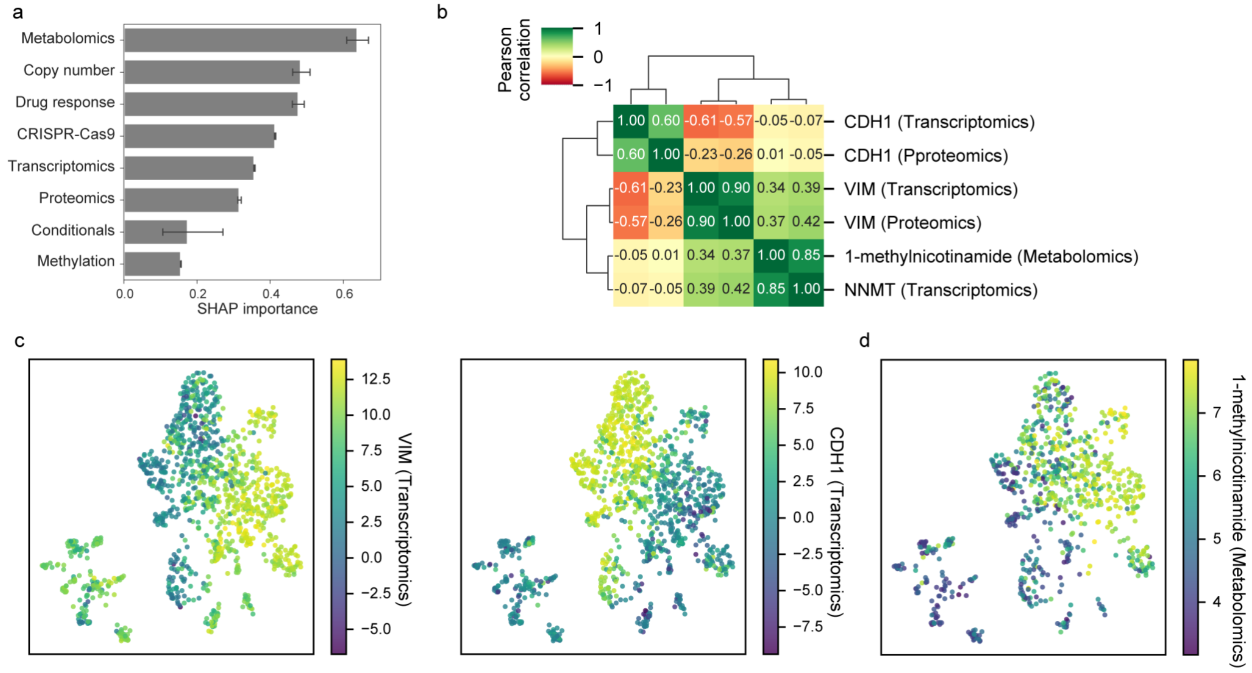

Supplementary Figure 7. *MOSA feature importance and joint latent space representations colored by specific markers,* ***a)*** *global feature importance grouped by omic datasets for constructing the joint latent space. Error bars represent 95% confidence intervals.* ***b)*** *correlation heatmap for some of the most predominant features of MOSA identified using the SHAP analysis.* ***c)*** *gene expression of canonical markers of epithelial mesenchymal transition (EMT), VIM (left) and CDH1 (right).* ***d)*** *similarly to b) but colored by the intracellular abundance of the 1-methylnicotinamide metabolite.*

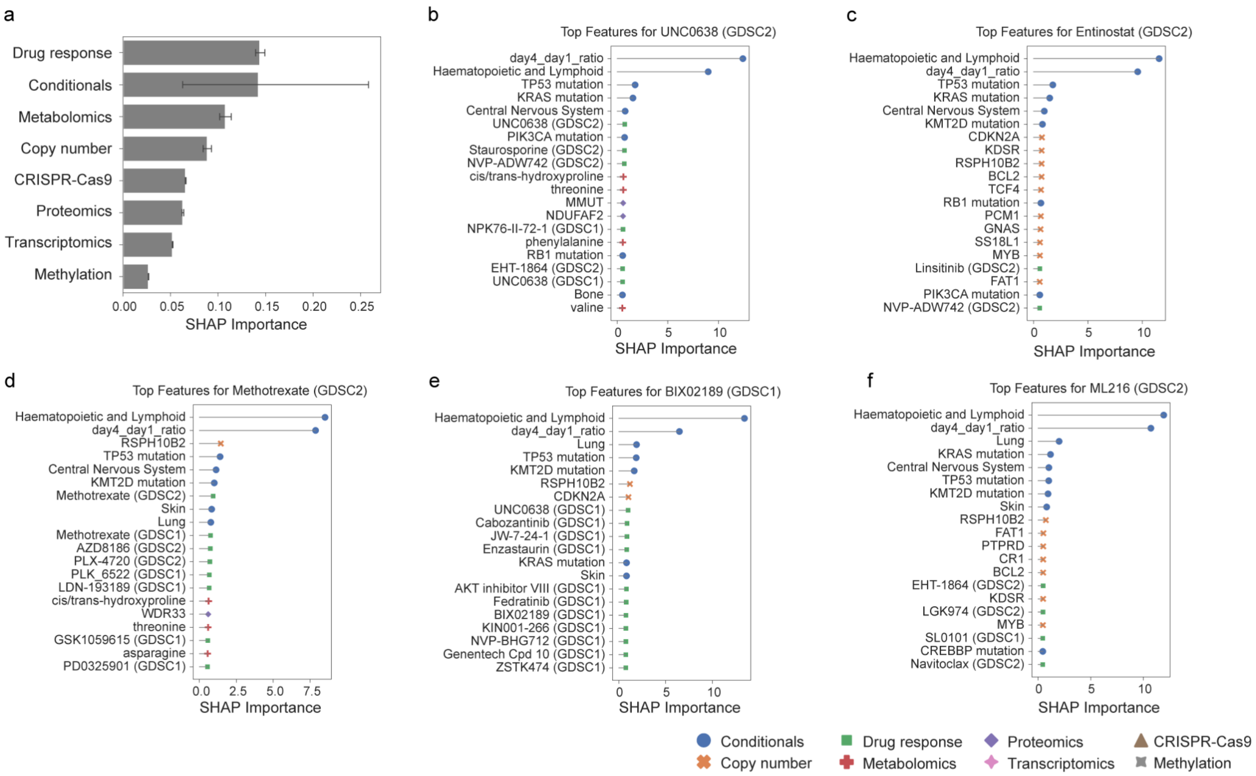

Supplementary Figure 8. *Feature importance calculated by SHAP values for the reconstruction of drug response.* ***a)*** *global feature importance grouped by omic datasets. Error bars represent 95% confidence intervals.* ***b-f****) top features for drugs that have the highest feature importance from 1-methylnicotinamide metabolite as shown in Figure 4b.*

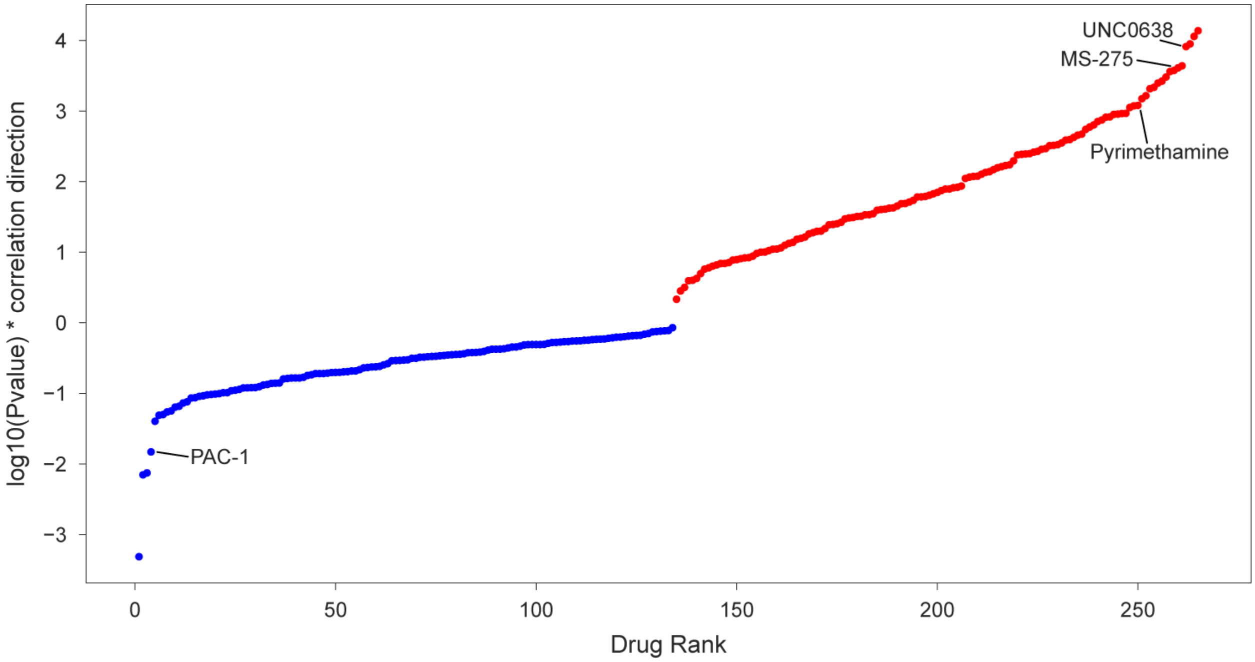

Supplementary Figure 9. *Drug rank correlation with nicotinate and nicotinamide metabolism. The plot shows the correlation of drug rank with log10(p-value) multiplied by the correlation direction. Blue dots represent drugs with negative correlations, indicating potential sensitivity, while red dots represent drugs with positive correlations, indicating potential resistance. Highlighted drugs, including PAC-1, Daraprim (Pyrimethamine), UNC0638, and Entinostat (MS-275), are annotated, showing their high rank as either sensitive or resistant drugs in relation to 1-methylnicotinamide.*

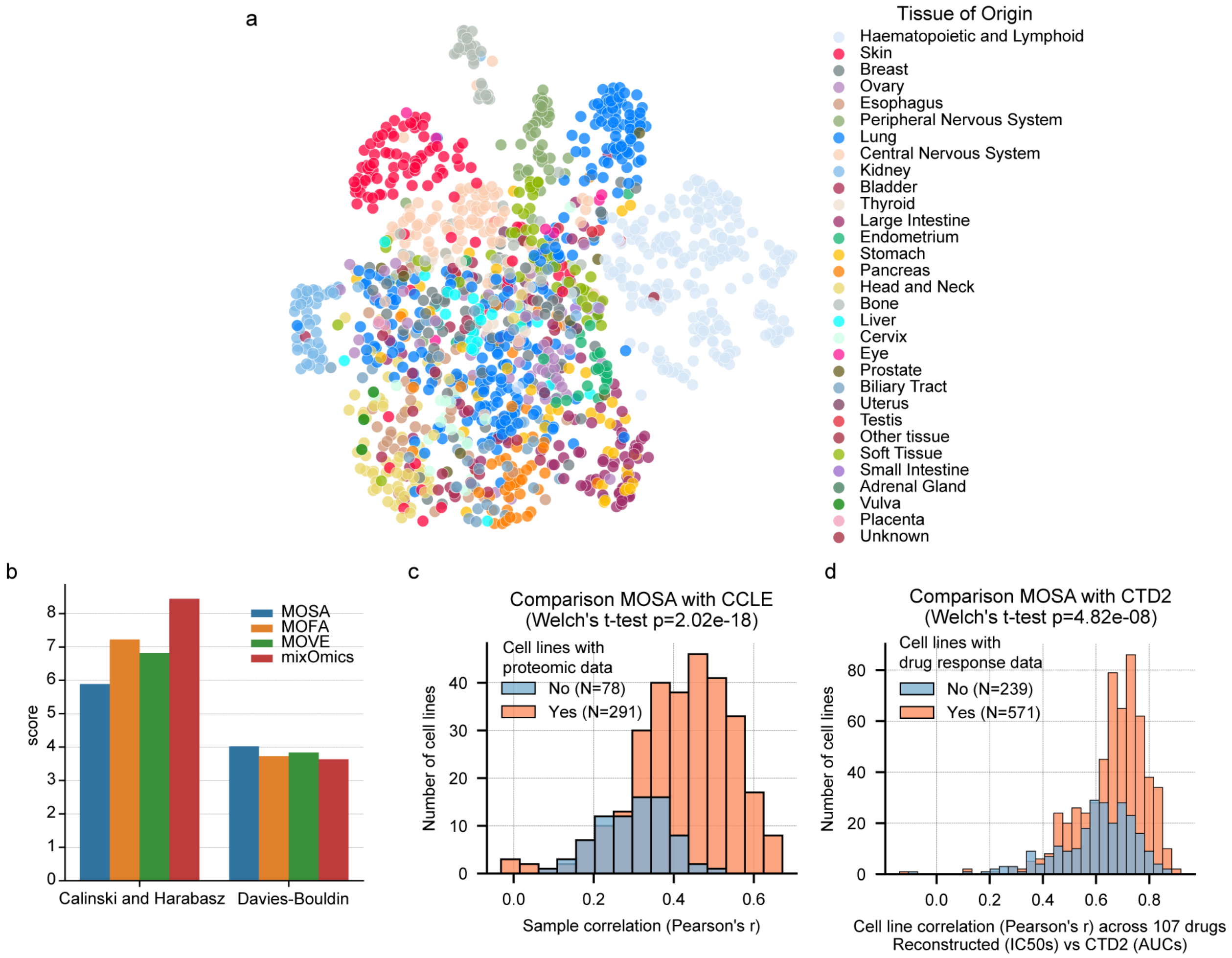

Supplementary Figure 10. *Evaluations of MOSA when view dropout is disabled.* ***a)*** *UMAP representation of the trained MOSA joint latent space. Similar to Figure 1c.* ***b)*** *comparison of cell line separations with MOFA, MOVE and mixOmics. Similar to Supplementary Figure 1d.* ***c)*** *data reconstruction for proteomic data. Similar to Figure 3a.* ***d)*** *data reconstruction for drug response data. Similar to Figure 3b.*
