# Supplementary material for: Synthetic augmentation of cancer cell line multi-omic datasets using unsupervised deep learning
Source: Nat Commun. 2024 Nov 29;15:10390. doi: 10.1038/s41467-024-54771-4 (PMC11607321; doi:10.1038/s41467-024-54771-4)
Supplement: Supplementary file 2 — Description of Additional Supplementary Files [file 41467_2024_54771_MOESM2_ESM.pdf]

Description of Additional Supplementary Files

Supplementary Data 1. Cancer cell line sample sheet and available datasets.

Supplementary Data 2. Multi-omics MOSA latent dimensions.

Supplementary Data 3. Conditional labels.

Supplementary Data 4. Omic feature masks.

Supplementary Data 5. Drug response and CRISPR-Cas9 10-fold cross-validation metrics.

Supplementary Data 6. Overview of the 16 multi-omics integration methods considered for the benchmark, including their limitations when applied to Cancer DepMap datasets.

Supplementary Data 7. Sample-wise proteomics CCLE correlation using different imputation methods. Two-sided Pearson correlation tests were used to assess the significance of the linear association between imputed and original protein intensities against the external CCLE dataset.

Supplementary Data 8. CRISPR-Cas9 gene essentiality associations with genetic alterations. One-sided log-ratio tests were used to assess the significance of genetic associations with CRISPR-Cas9 gene essentiality with the original dataset and the augmented MOSA dataset. False discovery rate (FDR) correction is applied using the Benjamini-Hochberg method to adjust for multiple comparisons.

Supplementary Data 9. Global level SHAP-based feature importance for explaining the latent space, aggregated across all the samples and latent dimensions.

Supplementary Data 10. Global level SHAP-based feature importance for explaining the drug response, aggregated across all the samples and drugs.
