# Supplementary material for: Synthetic augmentation of cancer cell line multi-omic datasets using unsupervised deep learning
Source: Nat Commun. 2024 Nov 29;15:10390. doi: 10.1038/s41467-024-54771-4 (PMC11607321; doi:10.1038/s41467-024-54771-4)
Supplement: Supplementary file 13 — Reporting Summary [file 41467_2024_54771_MOESM13_ESM.pdf]

nature portfolio

Corresponding author(s): Qing Zhong and Emanuel Gonçalves

Last updated by author(s): Oct 17, 2024



# Reporting Summary

Nature Portfolio wishes to improve the reproducibility of the work that we publish. This form provides structure for consistency and transparency in reporting. For further information on Nature Portfolio policies, see our Editorial Policies and the Editorial Policy Checklist.

## Statistics

For all statistical analyses, confirm that the following items are present in the figure legend, table legend, main text, or Methods section.

| n/a | Confirmed | |
|---|---|---|
| ☐ | ☒ | The exact sample size (*n*) for each experimental group/condition, given as a discrete number and unit of measurement |
| ☒ | ☐ | A statement on whether measurements were taken from distinct samples or whether the same sample was measured repeatedly |
| ☐ | ☒ | The statistical test(s) used AND whether they are one- or two-sided<br>*Only common tests should be described solely by name; describe more complex techniques in the Methods section.* |
| ☐ | ☒ | A description of all covariates tested |
| ☒ | ☐ | A description of any assumptions or corrections, such as tests of normality and adjustment for multiple comparisons |
| ☐ | ☒ | A full description of the statistical parameters including central tendency (e.g. means) or other basic estimates (e.g. regression coefficient) AND variation (e.g. standard deviation) or associated estimates of uncertainty (e.g. confidence intervals) |
| ☐ | ☒ | For null hypothesis testing, the test statistic (e.g. *F*, *t*, *r*) with confidence intervals, effect sizes, degrees of freedom and *P* value noted<br>*Give P values as exact values whenever suitable.* |
| ☒ | ☐ | For Bayesian analysis, information on the choice of priors and Markov chain Monte Carlo settings |
| ☒ | ☐ | For hierarchical and complex designs, identification of the appropriate level for tests and full reporting of outcomes |
| ☐ | ☒ | Estimates of effect sizes (e.g. Cohen's *d*, Pearson's *r*), indicating how they were calculated |

*Our web collection on statistics for biologists contains articles on many of the points above.*

## Software and code

Policy information about availability of computer code

| | |
|---|---|
| Data collection | All cancer cell line data used in this manuscript was collected from publicly available resources made available through the Cancer Dependency Map data portals. Assembled datasets and synthetic data generated in this manuscript are all made available through figshare repositories with details provided in the manuscript. |
| Data analysis | All analyses were performed using Python. All code is made available through a public GitHub repository (https://github.com/QuantitativeBiology/PhenPred) and a requirements file is provided with a list of all the required modules that could be used to systematically install all dependencies. |

For manuscripts utilizing custom algorithms or software that are central to the research but not yet described in published literature, software must be made available to editors and reviewers. We strongly encourage code deposition in a community repository (e.g. GitHub). See the Nature Portfolio guidelines for submitting code & software for further information.

## Data

Policy information about availability of data

All manuscripts must include a data availability statement. This statement should provide the following information, where applicable:

- Accession codes, unique identifiers, or web links for publicly available datasets
- A description of any restrictions on data availability
- For clinical datasets or third party data, please ensure that the statement adheres to our policy

All code is available at https://github.com/QuantitativeBiology/PhenPred. All data were assembled from the Cancer DepMap and synthetic datasets generated are

available for download at figshare:

● DepMap datasets:
○ https://doi.org/10.6084/m9.figshare.24420580
○ https://doi.org/10.6084/m9.figshare.24420598
● MOSA augmented datasets and latent representation:
○ https://doi.org/10.6084/m9.figshare.24562765
● MOSA feature importance:
○ https://doi.org/10.6084/m9.figshare.24473005
● MOFA multi-omics reconstruction and latent representation:
○ https://doi.org/10.6084/m9.figshare.24420631
● MixOmics multi-omics latent representation:
○ https://doi.org/10.6084/m9.figshare.25764408
● MOVE diabetes multi-omics reconstruction and latent representation:
○ https://doi.org/10.6084/m9.figshare.25764438

## Research involving human participants, their data, or biological material

Policy information about studies with human participants or human data. See also policy information about sex, gender (identity/presentation), and sexual orientation and race, ethnicity and racism.

| | |
|---|---|
| Reporting on sex and gender | Not relevant. |
| Reporting on race, ethnicity, or other socially relevant groupings | Not relevant. |
| Population characteristics | Not relevant. |
| Recruitment | Not relevant. |
| Ethics oversight | Not relevant. |

Note that full information on the approval of the study protocol must also be provided in the manuscript.

# Field-specific reporting

Please select the one below that is the best fit for your research. If you are not sure, read the appropriate sections before making your selection.

☒ Life sciences ☐ Behavioural & social sciences ☐ Ecological, evolutionary & environmental sciences

For a reference copy of the document with all sections, see nature.com/documents/nr-reporting-summary-flat.pdf

# Life sciences study design

All studies must disclose on these points even when the disclosure is negative.

| | |
|---|---|
| Sample size | Sample sizes were determined by the availability of cancer cell lines and the associated multi-omic datasets from the Cancer Dependency Map (DepMap). A total of 1523 cancer cell lines were included, for which at least two datasets were available. No sample-size calculation was performed as the study focuses on computational data analysis. |
| Data exclusions | For CRISPR-Cas9 gene essentiality, transcriptomic and methylomic feature reduction was performed to exclude lowly variable features. For gene essentiality, samples were scaled using essential and non-essential genes making their median per sample -1 and 0, respectively. Never essential genes were discarded, i.e., genes that do not have an essentiality profile lower than 50% of the median log2 fold-change of essential genes in at least one cell line were removed. For transcriptomics and methylomics, a standard deviation filter was applied. By taking the standard deviation of all genes across samples, a Gaussian mixture model (k=2) was fitted, identifying lowly variable genes and the rest. A standard deviation threshold was defined as the rightmost intercept of the two Gaussian distributions (Supplementary Figure 2a), and any gene with a standard deviation lower than that was discarded. Moreover, for the proteomic, drug response, metabolomic and CRISPR-Cas9 datasets, any feature with a missing rate higher than 85% was discarded. |
| Replication | The findings were validated using independent datasets for proteomics (CCLE), drug response (GDSC and CTD2), and transcriptomics. A 10-fold cross-validation strategy was applied to assess the reproducibility of the MOSA model across multiple omics layers. |
| Randomization | Data were randomly split for cross-validation purposes. The randomization was stratified by hematopoietic and lymphoid cell lines to ensure balanced representation across cell line types. |
| Blinding | Blinding was not applicable to this study, as all analyses were conducted using publicly available in vitro data from cancer cell lines. |

# Reporting for specific materials, systems and methods

We require information from authors about some types of materials, experimental systems and methods used in many studies. Here, indicate whether each material, system or method listed is relevant to your study. If you are not sure if a list item applies to your research, read the appropriate section before selecting a response.

## Materials & experimental systems

| n/a | Involved in the study |
| --- | --- |
| ☒ | ☐ Antibodies |
| ☐ | ☒ Eukaryotic cell lines |
| ☒ | ☐ Palaeontology and archaeology |
| ☒ | ☐ Animals and other organisms |
| ☒ | ☐ Clinical data |
| ☒ | ☐ Dual use research of concern |
| ☒ | ☐ Plants |

## Methods

| n/a | Involved in the study |
| --- | --- |
| ☒ | ☐ ChIP-seq |
| ☒ | ☐ Flow cytometry |
| ☒ | ☐ MRI-based neuroimaging |

# Eukaryotic cell lines

Policy information about cell lines and Sex and Gender in Research

| | |
| --- | --- |
| Cell line source(s) | Cancer cell lines used in this study were sourced from the processed data from Cancer Dependency Map (DepMap) and include a diverse range of cell lines representing various tissue types. The sex of the cell lines was not a primary variable in this study, though it is provided as metadata in the DepMap repository for reference. |
| Authentication | Cell lines used in this study were sourced from the Cancer Dependency Map (DepMap). All DepMap cell lines undergo quality control measures, including cell line authentication prior to data release. |
| Mycoplasma contamination | All cell lines used in this study were verified to be free of mycoplasma contamination by the Cancer Dependency Map (DepMap), which routinely tests for mycoplasma contamination as part of its quality control process. |
| Commonly misidentified lines (See ICLAC register) | No commonly misidentified cell lines were used in this study. All cell lines were obtained from the Cancer Dependency Map (DepMap), which verifies and authenticates cell line identities. |

# Plants

| | |
| --- | --- |
| Seed stocks | *Report on the source of all seed stocks or other plant material used. If applicable, state the seed stock centre and catalogue number. If plant specimens were collected from the field, describe the collection location, date and sampling procedures.* |
| Novel plant genotypes | *Describe the methods by which all novel plant genotypes were produced. This includes those generated by transgenic approaches, gene editing, chemical/radiation-based mutagenesis and hybridization. For transgenic lines, describe the transformation method, the number of independent lines analyzed and the generation upon which experiments were performed. For gene-edited lines, describe the editor used, the endogenous sequence targeted for editing, the targeting guide RNA sequence (if applicable) and how the editor was applied.* |
| Authentication | *Describe any authentication procedures for each seed stock used or novel genotype generated. Describe any experiments used to assess the effect of a mutation and, where applicable, how potential secondary effects (e.g. second site T-DNA insertions, mosiacism, off-target gene editing) were examined.* |
